# Public Health Department

**Published:** 1900-03

**Authors:** 


### Public Health Department.

[Dr. R. H. Harrison, Chairman Committee on Medical Legislation, will submit the following draft of a bill at the Waco meeting of the Texas State Medical Association, April 24th inst.—Ed.]

AN ACT to provide a uniform system of protection for the public health and the preservation of a record of the vital statistics of the State of Texas.

ARTICLE I.

SECTION 1. Be it enacted by the Legislature of the State of Texas:

That it shall be the duty of the Governor, and he is hereby authorized and empowered to select and appoint, by and with the advice and consent of the Senate, as soon as practicable after the passage of this act, from the most skillful physicians of the State, one who is a regular graduate of medicine, and who from experience and continuous practice of his profession shall be familiar with the various types of contagious, infectious, and preventable diseases, to be the State Health Officer.

Said State Health Officer shall be ex-officio president and chief executive officer of a State Board of Health hereinafter provided for and the official sanitary advisor of the government. He shall have charge of and superintend the administration of all quarantine service and disinfecting stations for the protection of the State from imported contagious and infectious diseases.

He shall also direct the investigation of the origin and nature of all epidemic and endemic diseases developed in any section of the State, and exercise a general supervision over the sanitary measures necessary to abate or stamp out any such epidemic diseases.

SEC. 2. It shall also be the duty of the Governor to appoint, as before, of the most skillful members of the medical profession, one who is a regular graduate in medicine, who is an expert in chemistry, microscopy and bacteriology, who shall be the pathologist of the Board; and another who is a graduate in veterinary medicine and surgery, who, together with the Attorney-General, shall constitute the State Board of Health; and said Board so constituted shall be charged with the duty of investigating the causes and character of epidemic and endemic diseases, and the means of preventing or arresting them wherever they occur within the limits of the State; of observing the influence of climate, locality, occupation, habits, temperature, rainfall, etc., in the production of disease or perpetuation of health; and all other matters of sanitary interest or scientific importance, bearing in any wise upon the protection of public health; of all of which they shall report fully to the Governor at such times and in such manner and form as may be required of other departments of the government.

SEC. 3. The Governor may, in his discretion, in cases of emergency, or where extraordinary outbreaks of disease may seem to require it, appoint a supplemental or advisory board, consisting of one member, qualified as above, from each supreme judicial district in the State, who shall be subject to the call of the president for purposes of investigation or consultation, as the case may be.

SEC. 4. The State Health Officer and president of the Board as above described shall appoint from the medical profession a regular graduate, who shall be secretary, whose duty is shall be to keep a record of the transactions of the Board and to receive and reply, under direction of the president, to all communications addressed to it.

SEC. 5. The secretary as above appointed shall also prepare, under the direction of the Board, all necessary blank forms for the acquisition and record of a comprehensive system of vital statistics of the State.

ARTICLE II.

SECTION 1. All county physicians now provided for by law shall be ex-officio county health officers and presidents of county boards of health for their respective counties, to be composed of the clerk of the county court and the county attorney, whose duty it shall be to investigate the character and causes of any epidemic or endemic diseases which may be developed within their respective counties, and the means of arresting or preventing them. They shall also observe the influence of climate, locality, occupation, habit, temperature, rainfall, etc., in connection with the prevailing types of diseases in their several counties; and report thereof semi-annually or oftener to the State Board of Health, if required.

SEC. 2. Said county health officer, as the executive head of the county board of health so constituted, shall superintend the collection of the vital statistics of the county, and the clerk of the county court shall keep a record of the same in connection with and supplemental to the record of marriages now provided for by law.

SEC. 3. It shall also be the duty of such county health officer to render advisory services to the county and corporate authorities within their jurisdiction on all questions involving the public health whenever called upon by such authorities. It shall likewise be their duty to attend, in person, or by their professional representative, upon all paupers, prisoners or other patients with which said county or corporate authorities may be chargeable; to inspect county and corporate jails and prisons; to examine the subjects of inquests in alleged cases of insanity; and to aid coroners and other legal authorities in the discharge of their duties by making post-mortem examinations or chemical analysis when necessary, for all of which said county or corporate authorities shall allow a fair compensation, according with the customary fee bill of physicians in similar cases, to be paid out of the county or corporate treasury as other obligations of said county or corporations are.

SEC. 4. In case of the prevalence of an epidemic in any county, or under other circumstances, when the legally constituted authorities of a county or corporation deem it necessary, they may make a special engagement with the county health officer to render special services for the protection of the public health, upon such terms and conditions as the parties may agree to.

SEC. 5. The county health officer as above indicated may be removed by the State Board of Health and a substitute appointed if found incompetent or negligent of his duty.

ARTICLE III.

SECTION 1. All physicians, surgeons and accouchers, who may attend at the birth of a child, or in the absence of any such attendants, any other party having knowledge of the same, shall report the fact to the clerk of the county court, together with the race to which it belongs, its sex, and whether legitimate or otherwise, still born or alive, within ten days after said birth occurs, under a penalty of five dollars to be collected as other fines for misdemeanors are.

SEC. 2. All physicians, surgeons, accouchers, coroners, or, in the absence of any of these, any other person who shall become cognizant of a death shall report the same [the cause of death], together with the race, sex, residence (whether alien or a citizen) to the clerk of the county court within ten days after the occurrence, under a penalty of not less than five nor more than fifty dollars, to be recorded as a part of the vital statistics of the county and State.

ARTICLE IV.

SECTION 1. The office of the State Board of Health shall be in the capitol at Austin, and it shall be supplied with all necessary material as other departments of the State government are supplied.

ARTICLE V.

SECTION 1. The president of the State Board of Health (State Health Officer) shall devote his entire attention to the sanitary affairs of the State to the exclusion of any other avocation whatever, and shall be allowed a salary of four thousand dollars per annum, and traveling expenses, to be paid out of said treasury as other salaries are. [How about the salaries of Secretary, Chemist and Veterinarian?—ED.]

ARTICLE VI.

This law shall take effect and be enforced from and after its passage, and all laws and parts of laws in conflict therewith are hereby repealed.

